# A Litany for Doctors

**Published:** 1913-11

**Authors:** 


A Litany For Doctors. From too few patients, and from too many patients; from hypodermic syringes that won't work; from book agents; from consultants who steal our cases; from rheumatism; from collecting agencies; from stupid nurses; from people who are going to pay for visit next Saturday night; from tire troubles and Christian Scientists—good Lord, deliver us.

From the people who begin their letters to us, "Dear Sir"; from static machines in damp weather; from boils on the back of the neck; from debts and detail men; from anti-vivisectionists; from nurses who know more than we do; from "cures" for tuberculosis; from "text-book" papers; from incurable cases of imaginary disease; from Bernard McFaddists; from tag days; from new methods for administering salvarsan; from "automobile" fractures; from infant foods; from anti-vaccinationists; from nature curers; from Immanuel Movers and the *treponema pallida*—good Lord, deliver us.

From the people who call us "Doc"; from malpractice; suits and dead beats; from gossips; from over-grateful female patients; from pretty nurses and jealous wives; from the doctor who succeeds us in a case; from the "wrong number" mistake; from consultations by telephone; from the counter-prescribing druggist; from lawyers and dentists; from the man who wants us to help his lady friend out of trouble; from calls at 2 A. M.; from shoulder presentations; from optometrists and engine trouble; from the man who "cannot add anything to the paper, but merely wants to compliment the essayist"; from meta-amidopenylparamethoxychinolin; from New Thoughters and mining stocks; from breaking catgut; from neurasthenics; from "the sponge we left behind us"; and from the dangers of tricresol 0.4 per cent.—good Lord, deliver us. Amen.—R. R. in *Lancet-Clinic.*

